# Cost-effectiveness of a stepped care program to prevent depression among primary care patients with diabetes mellitus type 2 and/or coronary heart disease and subthreshold depression in comparison with usual care

**DOI:** 10.1186/s12888-021-03367-z

**Published:** 2021-08-13

**Authors:** S. E. M. van Dijk, A. D. Pols, M. C. Adriaanse, H. W. J. van Marwijk, M. W. van Tulder, J. E. Bosmans

**Affiliations:** 1 Department of Health Sciences, Faculty of Science, Vrije Universiteit Amsterdam, Amsterdam Public Health research institute, De Boelelaan 1085, 1081 HV Amsterdam, the Netherlands; 2 Department of General Practice and Elderly Medicine and the Amsterdam Public Health research institute, VU University Medical Centre, Amsterdam, The Netherlands; 3 Department of Primary Care and Public Health Medicine, Brighton, UK; 4 Sussex Medical School, Brighton, UK

**Keywords:** Randomized controlled trial, Depression, Prevention, Cost-effectiveness analysis, Diabetes mellitus type 2, Cardiovascular disease

## Abstract

**Background:**

Patients with diabetes mellitus type 2 (DM2) and/or coronary heart disease (CHD) are at high risk to develop major depression. Preventing incident major depression may be an important tool in reducing the personal and societal burden of depression. The aim of the current study was to assess the cost-effectiveness of a stepped care program to prevent major depression (Step-Dep) in diabetes mellitus type 2 and/or coronary heart disease patients with subthreshold depression in comparison with usual care.

**Methods:**

An economic evaluation with 12 months follow-up was conducted alongside a pragmatic cluster-randomized controlled trial from a societal perspective. Participants received care as usual (*n* = 140) or Step-Dep (*n* = 96) which consisted of four sequential treatment steps: watchful waiting, guided self-help, problem solving treatment and referral to a general practitioner. Primary outcomes were quality-adjusted life years (QALYs) and cumulative incidence of major depression. Costs were measured every 3 months. Missing data was imputed using multiple imputation. Uncertainty around cost-effectiveness outcomes was estimated using bootstrapping and presented in cost-effectiveness planes and acceptability curves.

**Results:**

There were no significant differences in QALYs or depression incidence between treatment groups. Secondary care costs (mean difference €1644, 95% CI €344; €3370) and informal care costs (mean difference €1930, 95% CI €528; €4089) were significantly higher in the Step-Dep group than in the usual care group. The difference in total societal costs (€1001, 95% CI €-3975; €6409) was not statistically significant. The probability of the Step-Dep intervention being cost-effective was low, with a maximum of 0.41 at a ceiling ratio of €30,000 per QALY gained and 0.32 at a ceiling ratio of €0 per prevented case of major depression.

**Conclusions:**

The Step-Dep intervention is not cost-effective compared to usual care in a population of patients with DM2/CHD and subthreshold depression. Therefore, widespread implementation cannot be recommended.

**Trial registration:**

The trial was registered in the Netherlands Trial Register (NTR3715).

**Supplementary Information:**

The online version contains supplementary material available at 10.1186/s12888-021-03367-z.

## Background

Major depression is a common mental health problem in primary care that yearly affects approximately 3–9% of the general population [1, 2], and about 10–14% of all patients consulting their general practitioner (GP) [3] over a year. Currently, major depression ranks fourth in the list of diseases causing most disability as measured in disability adjusted life years (DALYs) in industrialized countries [4, 5]. It is expected that in 2030, depression will be the second largest contributor to DALYs globally [6]. Major depression is even more common in patients with type 2 diabetes mellitus (DM2) and/or coronary heart disease (CHD) with 12 month incidence rates ranging between 10 and 20% [7, 8]. Moreover, this combination of disorders leads to a substantial additional health challenge, as it is associated with a higher burden of disease resulting in reduced quality of life [9–11], adverse health outcomes [9–12], higher productivity losses [13–15] and higher health care use [16–18] compared to only having depression or DM2 and/or CHD [9–12, 16, 19].

Research has shown that the treatment success of major depression is low [20, 21]. Thus, preventing major depression is considered as a viable strategy to reduce its personal and economic burden. Considering the higher prevalence and chronicity of depression in patients with DM2 and/or CHD compared to the general population, prevention in this patient group could greatly reduce the personal and economical burden [22].

Subthreshold depression, i.e., the presence of depressive symptoms without fulfilling the criteria for major depressive disorder, is the most important predictor of major depression [23]. More than 40% of all DM2 and/or CHD patients with subthreshold depression will develop a major depressive disorder within two years [24, 25]. Moreover, subthreshold depression itself is associated with increased productivity losses [26] and health care utilization, and lower quality of life [27, 28].Therefore, treating subthreshold depression might be an effective strategy to prevent depression and the burden associated with depressive symptoms.

Previous research has shown that psychological interventions aimed to reduce subthreshold depression may prevent incident major depression [29]. Especially promising are interventions that are delivered in a stepped care format in which upscaling to more extensive treatment is only done when depressive symptoms remain present [30]. Also, stepped care may prevent incident major depression in older patients and patients with chronic diseases who also experience subthreshold depression, but the results are mixed at best [31–36]. Previous studies have shown that stepped care can be effective to treat major depression in patients with DM2 and/or CHD [37]. However, whether stepped care is cost-effective in preventing major depression in this population is unknown.

Economic evaluations of stepped care programs to prevent major depression are scarce. However, insight into the investments needed per unit of gained effect are urgently needed to decide whether the healthcare benefits stepped care possibly offers are affordable [38]. Especially healthcare policy makers who need to decide about the best way to spend the scarce resources available for healthcare have an urgent need for such information [38]. Therefore, in this study we assessed the cost-effectiveness of Step-Dep, a stepped care program to prevent major depression in primary care patients with DM2 and/or CHD who experience subthreshold depressive symptoms, in comparison with care as usual [39]. We hypothesized that Step-Dep would result in a reduction in the incidence of major depression and societal costs as compared to usual care.

## Methods

### Study design and setting

This economic evaluation was conducted from a societal perspective alongside the Step-Dep study; a cluster randomized controlled trial with measurements at baseline and 3, 6,9 and 12 months. Step-Dep was conducted between 2013 and 2015. The clusters consisted of 27 primary care centers in the Netherlands with 53 general practitioners (GPs) and 128,280 enlisted patients. The total number of enlisted patients per center ranged between 2000 and 8000. Centers were recruited through research networks of general practitioners. The Step-Dep intervention consisted of a stepped care program to prevent major depression in DM2/CHD patients with subthreshold depressive symptoms. To resemble daily practice as much as possible, the Step-Dep intervention was implemented by practice nurses who were already working at the participating primary care center. Details of the Step-Dep study are described elsewhere [39].

The study was performed in accordance with the declaration of Helsinki (2008) and the Dutch Medical Research involving Human Subjects Act (WMO). The protocol was approved by the medical ethics committee of the VU University Medical Centre (NL39261.029.12, registration number 2012/223), and registered in the Dutch Trial Register (NTR3715 http://www.trialregister.nl/trialreg/admin/rctview.asp?TC=3715).

### Randomization & blinding

Randomization was done at the level of the participating primary care centers to avoid contamination between the treatment groups. After consent to participate, a primary care center was randomly allocated to either the intervention group or the care as usual group by an independent statistician using a computer-generated list of random numbers. The statistician was blinded for the location and other characteristics of the primary care centers. Centers were stratified for size (2000 to 5000 patients or > 5000 patients).

### Participant selection

Patients could participate when they were diagnosed with DM2 and/or CHD, and were experiencing subthreshold depressive symptoms Patient Health Questionnaire-9 (PHQ-9) [40] score of 6 or more [41, 42], without meeting the criteria for a major depressive disorder according to the Mini International Neuropsychiatric Interview (MINI) [43]. Patients who were potentially eligible were selected by constructing a computer-generated list of all patients with an International Classification of Primary Care (ICPC) diagnosis of DM2 and/or CHD in the electronic patient record system (Additional file 1). Patients were included by the GP if they were considered to have cognitive impairment, psychotic illness or terminal illness, antidepressant medication use, loss of a significant other in the past 6 months, history of suicide attempts, visual impairment, or otherwise not being able to complete written questionnaires or visit the primary care center. Potentially eligible patients were invited by their GP to participate in the study and to fill out a PHQ-9 form to screen for depressive symptoms. Patients with a PHQ-9 score of six or higher were contacted within 2 weeks by trained research assistants after giving informed consent for a telephone interview. The research assistants administered the Mini International Neuropsychiatric Interview (MINI) to rule out current major depressive disorder. All patients who had a PHQ-9 score of 6 or more, did not meet the criteria for major depression according to the MINI, and provided informed consent were included.

### Intervention

The Step-Dep intervention was added onto usual care and comprised four sequential treatment steps with increasing intensity and a duration of 3 months each. After each step, the practice nurse evaluated depressive symptoms using the PHQ-9. The next step was initiated when a participant continued to experience subthreshold depression (PHQ-9 > = 6), otherwise watchful waiting was continued. When subthreshold depression (PHQ-9 > = 6) recurred after any period of remission, the next sequential step a participant had not yet received was offered. Trained practice nurses acted as care manager and coordinated the Step-Dep intervention. Practice nurses were trained to use motivational interviewing techniques to coach participants and to implement problem solving treatment (PST), and worked together with the GP.

Step 1 was a period of watchful waiting, which started with an introduction by the practice nurse. In this step, no active interventions were implemented by the practice nurse besides monitoring of depressive symptoms.

In step 2 a guided self-help course was offered [44]. The practice nurse monitored progress of the participants during routine phone calls every other week.

Step 3 consisted of PST provided by the practice nurse focusing on practical skill building [45, 46]. PST consisted of a maximum of 7 sessions during 12 weeks.

In step 4, the participant was referred to the GP when subthreshold depression was still present after completing the first 3 steps, and the participant still experienced a need for care. This step was also initiated at any time during the program, when a participant was diagnosed with major depressive disorder or had suicidal thoughts.

Participants in the care as usual condition had unrestricted access to usual care as provided by their GP. GPs and practice nurses in usual care centers did not receive any additional training or information about the Step-Dep intervention. In general, GPs and practice nurses follow Dutch guidelines for depression if they suspect depressive symptoms. The Dutch guidelines for depression recommend psychoeducation and short-lasting psychological support for depressive complaints, psychotherapy or antidepressants for major depressive disorder, and psychotherapy and antidepressants for severe major depressive disorder [47].

### Effects

All participants received web-based questionnaires at baseline, and at 3, 6, 9, and 12 months of follow up. When participants preferred hard copies, the questionnaires were sent by mail to the home address of the participant.

In the economic evaluation, the primary effect outcomes were 12-month cumulative incidence of major depression and Quality-Adjusted-Life-Years (QALYs). The cumulative incidence of major depression was assessed using the Dutch version of the MINI during telephone interviews that were administered by trained research assistants at 6 and 12 months [48]. Quality of life was measured using the EuroQol-5D-5L (EQ-5D-5L) at 3, 6, 9, and 12 months follow up [49]. The EQ-5D health states were converted to utility scores using the Dutch EQ-5D tariff [50]. Utility scores represent the relative desirability of a health state and are anchored at 0 (death) and 1 (best possible health related quality of life) [50]. QALYs were calculated by multiplying the utilities with the amount of time a participant spent in a particular health state. Transitions between health states were linearly interpolated.

Secondary outcomes were severity of depressive symptoms and perceived recovery from depressive symptoms. Severity of depressive symptoms was measured with the Dutch version of the PHQ-9 (range 0–27) with higher scores indicating more severe depression [51]. The PHQ-9 is widely used to measure depressive symptoms, and was previously validated in patients with chronic medical illnesses [52]. To indicate to what extent participants considered themselves to be recovered from depressive symptoms, they completed a 7-point Likert scale ranging from 1 (complete recovery) to 7 (worse than ever) with a score of 4 indicating no perceived change. For the purpose of the current study, the scores on this scale are dichotomized with scores 1–3 indicating recovery and scores 4–7 indicating no recovery.

### Costs

Costs were measured from a societal perspective, which means all costs were considered regardless of who pays for them [39]. Costs were converted to 2014 if necessary using Dutch consumer price indexes [53].

#### Intervention costs

To calculate intervention costs, a bottom-up micro-costing approach was used. More specifically, for each participant who participated in the intervention, the costs were estimated based on the treatment steps that were initiated for each participant [54]. The intervention costs consisted of all costs that were associated with implementation and execution of the Step-Dep program including the time the practice nurses spent preparing for consultations, providing care and registering provided care; the costs associated with the allocation of practice space during visits at the primary care center; the telephone costs for making appointments and guiding the self-help course; materials used for the self-help course and PST; and the costs of consultations with the GP.

#### Health care utilization costs

To measure healthcare utilization, an adapted version of the TIC-P questionnaire was used [55] which was administered every 3 months. Participants reported on the health care they received (e.g., GP, physiotherapist, medical specialist, psychologist, hospitalization and complementary care), medication (prescription and over the counter) they used, and informal care (by informal caregivers such as relatives, friends, and neighbors) they received. Dutch standard cost prices were used when available [56]. Otherwise, we used prices from Dutch professional organizations. Medication was valued using standard prices that were published by the Royal Dutch Society for Pharmacy [57].

#### Productivity costs

Costs of productivity losses were also included. Absenteeism was measured using a single item in which participants reported the number of sick days during the last 3 months. Costs per sick day were calculated by using the mean wage per hour for every age group for men and women separately [56] and multiplying this by the mean number of hours a participant worked per day. The costs associated with absenteeism were calculated using both the friction costs and the human capital approach [56]. As none of the participants reported a consecutive sickness absence of more than the friction period of 23 weeks, both approaches resulted in the same estimation of absenteeism costs and are, therefore, not reported separately. Presenteeism was assessed using a single question in which participants reported on the number of hours they would need to work extra to compensate for productivity losses due to sickness while present at work. Costs associated with presenteeism were calculated by multiplying this number of hours with the mean wage per hour for the age group and gender of the participant [56].

### Statistical analyses

All analyses were carried out according to the intention-to-treat principle using STATA version 12SE. Missing data were imputed using multiple imputation according to the Multiple Imputation by Chained Equations (MICE) algorithm [58]. Predictive Mean Matching was used to account for the skewed distribution of costs [59]. Variables that were associated with missing data, and variables that were associated with the outcomes, were identified and included in the imputation model. Also, all variables in the analysis model (costs, effects and potential confounders) were included. The number of imputed datasets was increased until the loss of information was less than 5% [59]. In total 10 datasets were imputed. The subsequent analyses were performed on each imputed dataset separately after which results were pooled using Rubins rules [60].

To estimate cost and effect differences, seemingly unrelated regression analyses were performed, since multilevel analyses were not necessary considering the small intra class correlation within primary care centers (7%). Incremental Cost Effectiveness Ratios (ICERs) were calculated by dividing the differences in total societal costs by the effect differences. Because the distribution of cost data is usually severely skewed to the right, statistical uncertainty surrounding the estimates was assessed using bias-corrected accelerated bootstrapping with 5000 replications. Subsequently, 95% confidence intervals surrounding differences in total costs and disaggregated costs were estimated. Bootstrapped cost-effect pairs were plotted in cost-effectiveness planes to graphically display the uncertainty surrounding the ICERs [61]. Furthermore, Cost-Effectiveness Acceptability Curves (CEACs) were estimated to depict the probability of an intervention being cost effective as compared to usual care for various maximum amounts of money decision makers are willing to pay for a unit of gained effect (i.e. ceiling ratio) [62].

The main analysis consisted of a fully adjusted model in which estimates were adjusted for baseline values, gender, age and other possible confounders: marital status, employment status, level of education, co-existence of DM2 and CHD, alcohol use, number of depressive episodes in history and age of onset of depression [63].

### Sensitivity analyses

To test whether the results of the main analysis were robust, two sensitivity analyses were performed. In the first sensitivity analysis, a crude model was estimated containing only the outcome and the group allocation, and no baseline values or confounders. In the second sensitivity analysis, a healthcare perspective was adopted [64]. In this perspective, only direct health care costs are taken into account (primary care, secondary care, intervention and medication costs).

## Results

### Participants

**Fig. 1:**
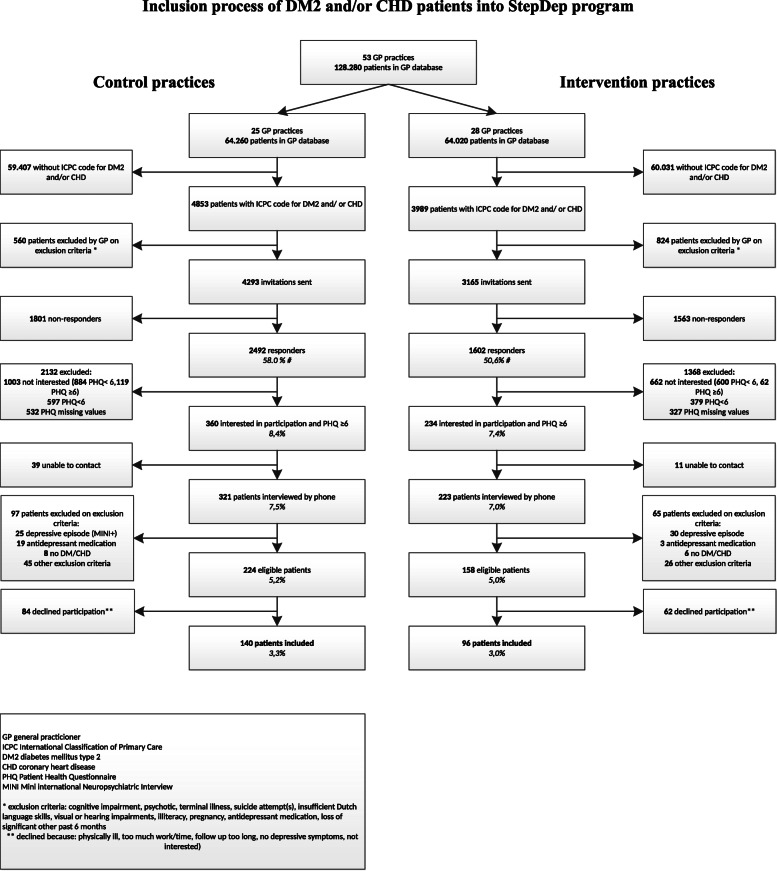
Flow of participants through the trial

Figure 1 visualizes the participant flow. In total, 96 participants were included in the intervention group and 140 in the usual care group. The uptake of the intervention was relatively low [63]. Only 25 of all 96 participants (26%) who were included in the intervention group proceeded to step 2, 9 participants (9%) to step 3 and 3 participants (3%) were referred to the GP (step 4) [63].

Baseline characteristics are summarized in Table 1. Of all participants, 70 (73%) in the intervention group and 92 (66%) in the care as usual group had complete cost and effect data for all time points. Only 26 participants (11%) dropped out of the study before follow up was complete and 48 participants (20%) missed one or more measurements without being completely lost to follow up. Participants with more different chronic medical illnesses, and who had lower quality of life at baseline were more likely to have missing data at some point of follow-up.

**Table 1:** Baseline characteristics of the participants assigned to the intervention group (Step-Dep) or usual care group (control)

Characteristics	Intervention (*n* = 96)	Care as usual (*n* = 140)
Female	42 (43.8)	65 (46.4)
Age, mean (SD)	67.8 (9.2)	67.3 (10.5)
Marital status		
Married/living together	55 (57.3)	67 (47.9)
Single/divorced/widowed	35 (36.5)	63 (45)
Not reported	6 (6.3)	10 (10.4)
Both parents born in the Netherlands	74/90 (82.2)	112/130 (86.2)
Rural residential area	42 (43.8)	57 (40.7)
Unemployed/sick	12/90 (13.3)	14/130 (10.8)
Level of education^1^		
Low	33 (34.4)	56 (40)
Average	22 (22.9)	38 (27.1)
High	35 (36.5)	36 (25.7)
Not reported	6 (6.3)	10 (7.1)
Diabetes Mellitus type 2 (DM2)	60 (62.5)	90 (64.3)
Coronary Heart Disease (CHD)	58 (60.4)	90 (64.3)
DM2 and CHD	22 (22.9)	40 (28.6)
Number of chronic diseases, median (25th -75th percentile)	3 (2–5)	3 (2–5)
DM2 treated with insulin or oral medication	42/57 (73.7)	64/83 (77.1)
CHD treated with chronic medication	46/54 (85.2)	65/85 (76.5)
Current smoker	16/90 (17.8)	23/129 (17.8)
Alcohol use above norm^2^	29/90 (32.2)	34 /129 (26.4)
Exercise under norm^3^	56/90 (62.2)	85/129 (65.9)
BMI, mean (SD)	29.4 (6.8)	28.5 (5.6)
EQ-5D-5L, mean (SD)	9,6 (2.9)	9,4 (2,9)
Locus of Control, mean (SD)	8.3 (4.2)	7.6 (4.1)
Social support, mean (SD)	35.8 (9.0)	36.7 9.5)
Dysthymia	6 (6.3)	7 (5.0)
Nr of depression in history		
0	35 (36.5)	65 (46.4)
1	14 (14.6)	11 (7.8)
2 or more	40 (41.7)	43 (30.7)
Not reported	7 (7.3)	21 (15)
Onset of depression after age of 55	38/89 (42.7)	63/121 (52.1)
PHQ-9 at baseline, mean (SD)	9.5 (3.1)	9.3 (3.2)
Depression HADS, mean (SD)	6.9 (3.9)	6.1 (3.7)
Anxiety HADS, mean (SD)	6.9 (3.7)	6.3 (3.9)

Figures are numbers (percentage) unless stated otherwise

*BMI* Body Mass Index, *EQ-5D-5L* Euroqol 5 dimensions 5 levels *CHD* coronary heart disease; *DM2* diabetes mellitus type 2; *HADS* Hospital Anxiety and Depression Scale; *PHQ-9* Patient Health Questionnaire-9 *NL* The Netherlands; *SD* Standard Deviation

^1^ Low level of education = did not finish high school; average level of education = finished high school and/or vocational education; high level of education = finished college or university

^2^ The norm for alcohol use is defined as drinking more than two standard units of alcoholic beverages on an average day for male participants. For female participants this norm is defined as drinking more than one standard unit of alcoholic beverages on an average day

^3^ The norm for healthy physical activity is defined as medium to high intensity exercise during 30 or more minutes on five or more days a week during a typical week

### Effects

Table 2 shows the effects in the two groups and the difference between them. The adjusted cumulative incidence of major depression was 13.4% in the intervention group and 11.6% in the usual care group. The difference was not statistically significant (adjusted mean difference 0.2%; 95% Confidence Interval (CI) -12%; 7%). The difference in QALYs between the intervention and usual care group was small and not statistically significant (mean difference 0.002, 95% CI -0.41; 0.46). Differences in change in depression severity and perceived recovery between the intervention and control group were also small and not statistically significant (Table 2).

**Table 2:** Mean crude health related costs per patient from a societal perspective and mean 12-month follow up

	Mean (SE) intervention	Mean (SE) usual care	Mean difference (95% CI)
Outcomes			
Cumulative incidence of depression	0.134 (0.013)	0.116 (0.019)	0.018 (− 0.077; 0.113)
QALY’s gained	0.709 (0.031)	0.708 (0.038)	−0.001 (− 0.479; 0.457)
Change in severity of depressive symptoms	−2.672 (0.416)	− 2.768 (0.630)	− 0.096 (− 1.454; 1.646)
Perceived recovery	0.569 (0.045)	0.572 (0.065)	0.027 (− 0.156; 0.162)
Costs			
Step-Dep intervention costs	€98 (€7)	€0 (€0)	€98 (€86; €112)
Primary care	€2310 (€307)	€3580 (€918)	€-1270 (€- 4369; €32)
Secondary care	€3509 (€658)	€1865 (€329)	€1644 (€344; €3370)
Informal care	€3711 (€868)	€1780 (€233)	€1930 (€528; €4089)
Medication	€997 (€132)	€736 (€66)	€261 (€34; €602)
Total direct costs from societal perspective	€10,625 (€1362)	€7961 (€1133)	€ 2664 (€-908; €6101)
Unpaid productivity	€5775 (€945)	€6889 (€931)	€-1114 (€-3497; €1454)
Absenteeism	€480 (€162)	€713 (€200)	€-316 (€-904; €168)
presenteeism	€356 (€161)	€672 (€217)	€-232 (€-762; €253)
Total indirect costs	€6611 (€1028)	€8274 (€994)	€ -1474 (€-3924; €1182)
Total societal perspective costs	€17,236 (€2137)	€16,235 (€1632)	€ 1001 (€-3975; €6409)

*CI* Confidence interval; *SE* Standard Error; *NHS* National Health Service (United Kingdom)

### Costs

In Table 2, the mean unadjusted costs per participant are presented. The costs of the intervention were 98 euro. Secondary care costs (mean difference €1644, 95% CI €344; €3370) and informal care costs (mean difference €1930, 95% CI €528; €4089) were statistically significantly higher in the intervention group than in the usual care group. Differences in costs for all other cost categories were not statistically significant. Total societal costs in the intervention group were higher than in the usual care group (mean difference €1001, 95% CI €-3975; €6409), but this difference was not statistically significant.

### Cost-effectiveness analyses

**Fig. 2:**
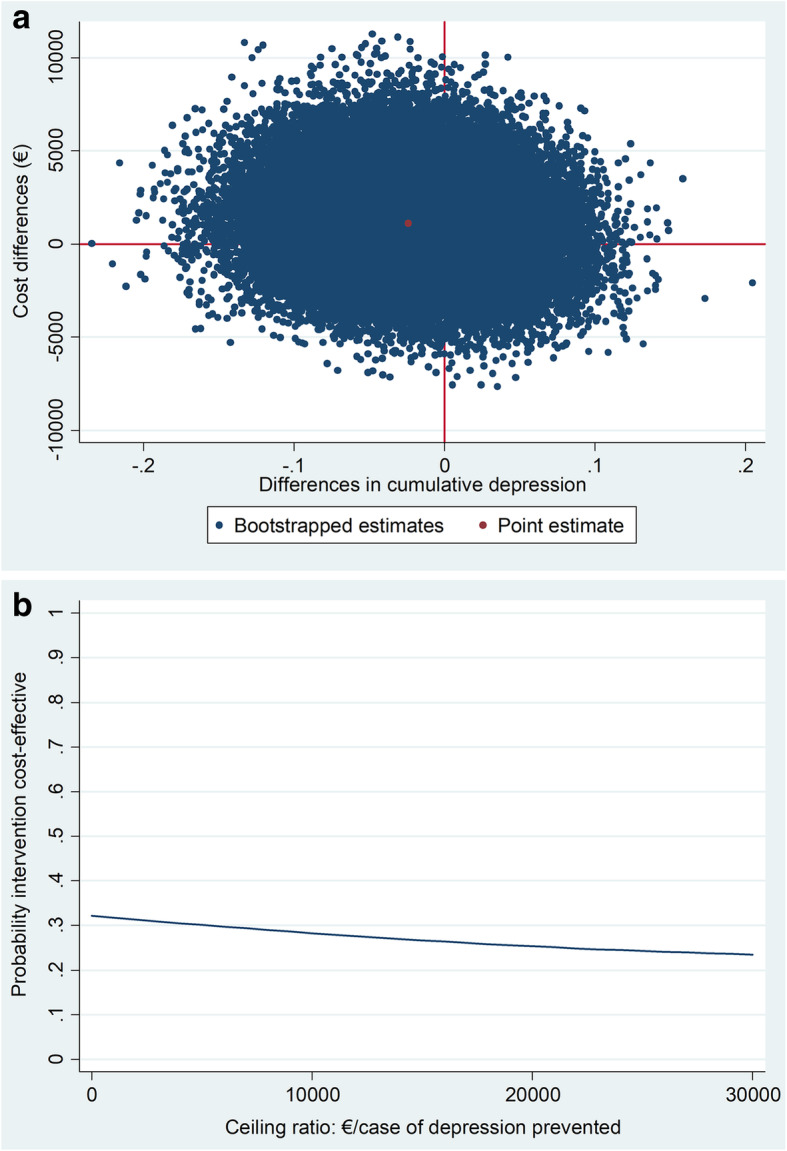
**a** Cost-effectiveness plane visualizing the uncertainty surrounding the incremental cost-effectiveness ratio for cumulative depression in the main analysis; (**b**) Cost-effectiveness acceptability curve visualizing the probability of the intervention being cost effective at different values of willingness to pay in Euros for cumulative depression in the main analysis

For the 12-month cumulative incidence of major depression, the ICER was − 46,802 €/prevented case of major depression, indicating that the intervention was more costly and less effective than usual care (Table 3). Although the majority of cost-effect pairs (49%) were situated in the NE quadrant, the CE plane shows that the cost-effect pairs were distributed across all 4 quadrants of the CE plane indicating that statistical uncertainty around the estimates is large (Fig. 2a). The CEAC shows that the maximum probability of the intervention being cost effective is 32% at a ceiling ratio of 0 €/prevented case of major depression (Fig. 2b). Because the intervention was less effective than usual care, the probability of cost-effectiveness decreased with higher ceiling ratios.

**Table 3:** Results of the cost-effectiveness analyses

Outcome	Mean cost difference (95% CI)	Mean effect difference (95% CI)	ICER	Distribution of the Cost-Effectiveness plane (%)			
				North-East	South-East	South-West	North-West
Main analyses^a^							
Cases of major depression prevented	€1125 (€-2942; €5343)	−0.024 (− 0.118; 0.070)	−46,802	19%	11%	21%	49%
QALYs (EQ-5D)	€919 (€-3133; €5145)	0.010 (−0.0269; 0.0474)	88,898	41%	30%	5%	24%
Severity of depressive symptoms (PHQ-9)	€923 (€-3151; €5073)	0.077 (−1.546; 1.701)	11,927	32%	23%	12%	33%
Perceived recovery	€1125 (€-2987; €5343)	0.011 (−0.156; 0.177)	107,353	38%	17%	14%	31%
Sensitivity analyses 1: Crude analyses^b^							
Cases of major depression prevented	€ 1001 (€-3543; €5852)	−0.018 (− 0.113; 0.077)	−55,779	20%	16%	20%	44%
QALYs (EQ-5D)	€ 1001 (€-3547; €5780)	0.001 (−0.457; 0.479)	939,235	26%	26%	9%	36%
Severity of depressive symptoms (PHQ-9)	€ 1001 (€-3577; €5797)	0.096 (−1.646; 1.454)	10,429	32%	23%	12%	33%
Perceived recovery	€ 1001 (€-3540; €5800)	−0.027 (− 0.162; 0.156)	− 373,720	32%	17%	19%	32%
Sensitivity analyses 2: analyses from NHS perspective^c^							
Cases of major depression prevented	€1422 (€-578; 3167)	−0.024 (− 0.118; 0.070)	−58,733	27%	3%	5%	65%
QALYs (EQ-5D)	€1369 (€-590; 3110)	0.010 (−0.0274; 0.0469)	140,088	60%	8%	2%	30%
Severity of depressive symptoms (PHQ-9)	€1398 (€-596; 3154)	0.077 (−1.541; 1.695)	18,174	49%	6%	3%	42%
Perceived recovery	€1422 (€-541; 3173)	0.0126 (−0.153; 0.179)	112,624	53%	4%	4%	39%

*CI* Confidence Interval; *MINI* Mini International Neuropsychiatric Interview; *EQ-5D-5L* EuroQo-5Dimensions 5 Levels; *PHQ-9* Patient Health Questionnarie-9; *NHS* National Health Service (United Kingdom)

^a^ Costs from a societal perspective are analyzed. All analyses are corrected for baseline values of costs from a societal perspective and the effect, gender, age and other possible confounders: marital status, employment status, level of education, co-existence of DM2 and CHD, alcohol use, number of depressive episodes in history and age of onset of depression

^b^ Costs from a societal perspective are analyzed. The analyses are not corrected for baseline values of costs and effects, nor for possible confounders

^c^ Costs from a NHS perspective are analyzed. All analyses are corrected for baseline values of costs from NHS perspective and the effect, gender, age and other possible confounders: marital status, employment status, level of education, co-existence of DM2 and CHD, alcohol use, number of depressive episodes in history and age of onset of depression

**Fig. 3:**
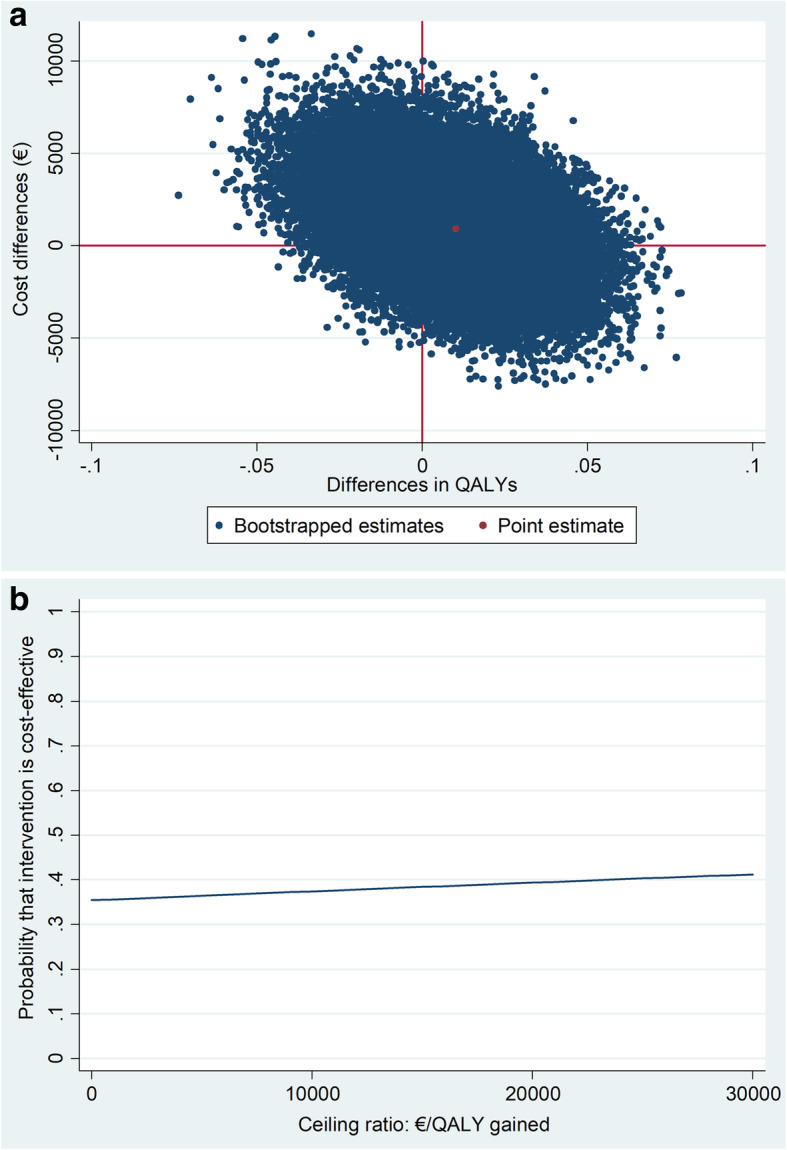
**a** Cost-effectiveness plane visualizing the uncertainty surrounding the incremental cost-effectiveness ratio for QALYs in the main analysis; (**b**) Cost-effectiveness acceptability curve visualizing the probability of the intervention being cost effective at different values of willingness to pay in Euros for QALYs in the main analysis

The cost-utility ratio was 88,898 €/QALY, indicating that €88,898 needs to be paid to gain one QALY in the intervention group as compared to the usual care group. Cost-effect pairs were distributed over all four quadrants of the CE plane, indicating substantial statistical uncertainty around costs and effects (Fig. 3a). The CEAC (Fig. 3b) shows that the maximum probability of the Step-Dep intervention being cost effective in the intervention group as compared to the usual care group is 41% at a ceiling ratio of 30,000 €/QALY.

The results of the analyses for severity of depressive symptoms are comparable to those for the cumulative incidence of depression (Table 3). The results of the analyses for perceived recovery are similar to the results for QALYs (Table 3).

### Sensitivity analyses

Results from the crude analyses were similar to the results of the main analyses. Using the healthcare perspective, the cost difference between the groups was larger than in the main analysis; €2664 instead of €1001 (unadjusted estimates; Table 2). Therefore, in these analyses the ICER estimates are larger and a greater proportion of cost-effect pairs are situated in the northern quadrants of the CE plane compared to the main analysis. Thus, from the healthcare perspective the stepped care program was even less cost-effective in comparison with usual care than from the societal perspective.

## Discussion

This study shows that the Step-Dep intervention for the prevention of major depression in primary care participants with DM2 and/or CHD and subthreshold depression is not cost-effective in comparison with usual care. The results of the current study are contrasting with previous studies that evaluated the cost-effectiveness of comparable stepped care interventions to prevent major depression [65] and to treat subthreshold depression [66]. The main difference with these previous studies is the lack of effectiveness of the Step-Dep intervention and the lower than expected risk of developing depression in both arms.

In the Dutch primary care system, participants with DM2 and/or CHD are seen regularly by their GP and/or practice nurse, and care givers are expected to ask participants about their mental health during these consultations. When a participant reports problems in this area, psychological care may be offered [67]. This may have diminished the contrast between the study groups. However, utilization rates of mental healthcare services and psychotropic medication in both groups were low, and did not differ much between groups (data not shown). In this study, secondary and informal health care costs were significantly higher in the intervention group than in the usual care group, while primary care costs were lower. Perhaps intervention participants focused on other aspects of their health than normally and shifted their attention to more different health domains, which could have led to different care seeking behavior outside primary care.

There is substantial evidence showing the effectiveness of stepped care interventions for the treatment of depression [68], although the evidence regarding cost-effectiveness is less convincing [69]. When looking at stepped care interventions for the prevention of depression, however, results are more conflicting. Studies among geriatric populations and visually disabled populations showed that stepped care reduced the incidence of depression [31–33], whereas other studies showed no effect [35, 36, 70]. Results on the cost-effectiveness of these interventions differed, with one study showing that stepped care was dominant over usual care [71], one study showing that stepped care was associated with health benefits at affordable costs [65], and one study showing that stepped care was not cost-effective [72]. Our study was in line with the studies showing that stepped care is not effective nor cost-effective in comparison with usual care. Possible explanations for these findings are that symptoms of depression may be overlapping with symptoms of DM2 and/or CHD and that the uptake of the stepped care intervention was low in our study.

This study has several strengths. First, to our knowledge, it is the first to evaluate the cost-effectiveness of a stepped care approach to prevent the onset of major depression in participants with DM2 and/or CHD with subthreshold depression. Second, there was a long follow-up period (12 months), while the number of dropouts was low (*n* = 26, 11%). This minimizes the chances of attrition bias. Third, the pragmatic design and implementation of the intervention maximizes the generalizability of the results to daily practice. Fourth, we evaluated the cost-effectiveness of the Step-Dep intervention from a societal perspective, taking in account all health-related costs, but also costs of informal care and lost productivity. This is more informative for decision makers in health care than narrower perspectives, since shifting of resources between different sectors becomes clear.

However, there are also some limitations to our study. First, blinding of caregivers and participants was not possible due to the nature of the intervention. Additionally, as with all cost-effectiveness studies, it is difficult to generalize the results outside of the researched health care system (the Dutch health care system in this case). Also, the implementation rate of the intervention was relatively low [63]. This was partly due to the improvement in depressive symptoms in both groups after inclusion making referral to more intensive treatment steps unnecessary. This is in contrast with the results of the process evaluation conducted alongside this study that showed that practice nurses considered the intervention beneficial for participants’ overall wellbeing [73]. Possibly, the need for care in the selected population was low, compromising the contrast between treatment groups. This may also mean that participants in the usual care group received more intensive treatment than in the intervention group. If this was the case, this should be reflected in the healthcare utilization cost in the usual care group, but these did not differ significantly between groups (data not shown). With a larger implementation rate, effects of the intervention might have been larger and costs higher. However, total costs could become lower when participants receive more adequate treatment for their depression, and thus seek less care elsewhere, for example in (more expensive) secondary care. Moreover, more effective treatment may be associated with reduced morbidity in the long term. Finally, the study’s power was based on the expected incidence rate of major depression. However, this incidence was much lower than expected. Additionally, because of the skewed distribution of costs larger sample sizes are generally needed than for clinical effects [74].

## Conclusions

Based on the current results, we conclude that the Step-Dep intervention is not cost-effective compared to usual care. The results of this study do not support widespread implementation of the Step-Dep intervention. However, it should be considered that treatment uptake was low and that the study was underpowered. Further research could show whether stepped care to prevent depression is more beneficial among the subgroup of people who express a need for treatment for their depressive symptoms.

## Supplementary Information

**Additional file 1.**

## Data Availability

The datasets generated and/or analyzed during the current study are not publicly available due to the inclusion of personal data, but are available from the corresponding author on reasonable request.

## References

[1] de Graaf R, Ten Have M, van Gool C (2012). Prevalence of mental disorders and trends from 1996 to 2009. Results from the Netherlands mental health survey and incidence Study-2. Soc Psychiatry Psychiatr Epidemiol.

[2] Kessler RC, Üstün TB. The WHO World Mental Health Surveys: Global Perspectives on the Epidemiology of Mental Disorders. New York: Cambridge University Press; 2008.

[3] Licht-Strunk E, van der Kooij KG, van Schaik DJ (2005). Prevalence of depression in older patients consulting their general practitioner in the Netherlands. Int J Geriatr Psychiatry.

[4] Üstün TB, Ayuso-Mateos JL, Chatterji S, Mathers C, Murray CJL (2004). Global burden of depressive disorders in the year 2000. Br J Psychiatry.

[5] Murray CJ, Vos T, Lozano R (2012). Disability-adjusted life years (DALYs) for 291 diseases and injuries in 21 regions, 1990–2010: a systematic analysis for the global burden of disease study 2010. Lancet.

[6] Mathers CD, Loncar D (2006). Projections of global mortality and burden of disease from 2002 to 2030. PLoS Med.

[7] Anderson RJ, Freedland KE, Clouse RE, Lustman PJ (2001). The prevalence of comorbid depression in adults with diabetes: a meta-analysis. Diabetes Care.

[8] Rudisch B, Nemeroff CB (2003). Epidemiology of comorbid coronary artery disease and depression. Biol Psychiatry.

[9] Moussavi S, Chatterji S, Verdes E, Tandon A, Patel V, Ustun B (2007). Depression, chronic diseases, and decrements in health: results from the world health surveys. Lancet.

[10] Ali S, Stone M, Skinner TC, Robertson N, Davies M, Khunti K (2010). The association between depression and health-related quality of life in people with type 2 diabetes: a systematic literature review. Diabetes Metab Res Rev.

[11] de Jonge P, Spijkerman TA, van den Brink RH, Ormel J (2006). Depression after myocardial infarction is a risk factor for declining health related quality of life and increased disability and cardiac complaints at 12 months. Heart.

[12] Katon W, Lin EH, Kroenke K (2007). The association of depression and anxiety with medical symptom burden in patients with chronic medical illness. Gen Hosp Psychiatry.

[13] Bielecky A, Chen C, Ibrahim S, Beaton DE, Mustard CA, Smith PM (2015). The impact of co-morbid mental and physical disorders on presenteeism. Scand J Work Environ Health.

[14] Egede LE (2007). Major depression in individuals with chronic medical disorders: prevalence, correlates and association with health resource utilization, lost productivity and functional disability. Gen Hosp Psychiatry.

[15] Ervasti J, Vahtera J, Pentti J, Oksanen T, Ahola K, Kivekäs T, Kivimäki M, Virtanen M (2014). The role of psychiatric, cardiometabolic, and musculoskeletal comorbidity in the recurrence of depression-related work disability. Depress Anxiety.

[16] Egede LE, Gebregziabher M, Zhao Y, Dismuke CE, Walker RJ, Hunt KJ, Axon RN (2015). Differential impact of mental health multimorbidity on healthcare costs in diabetes. Am J Manag Care.

[17] Bosmans JE, Adriaanse MC (2012). Outpatient costs in pharmaceutically treated diabetes patients with and without a diagnosis of depression in a Dutch primary care setting. BMC Health Serv Res.

[18] Dennehy EB, Robinson RL, Stephenson JJ, Faries D, Grabner M, Palli SR, Stauffer VL, Marangell LB (2015). Impact of non-remission of depression on costs and resource utilization: from the COmorbidities and symptoms of DEpression (CODE) study. Curr Med Res Opin.

[19] Ervasti J, Vahtera J, Pentti J, Oksanen T, Ahola K, Kivekäs T, Kivimäki M, Virtanen M (2015). Return to work after depression-related absence by employees with and without other health conditions: a cohort study. Psychosom Med.

[20] Andrews G, Issakidis C, Sanderson K, Corry J, Lapsley H (2004). Utilising survey data to inform public policy: comparison of the cost-effectiveness of treatment of ten mental disorders. Br J Psychiatry.

[21] Chisholm D, Sanderson K, Ayuso-Mateos JL, Saxena S (2004). Reducing the global burden of depression: population-level analysis of intervention cost-effectiveness in 14 world regions. Br J Psychiatry.

[22] Cuijpers P, Beekman AT, Reynolds CF (2012). Preventing depression: a global priority. Jama.

[23] Meeks TW, Vahia IV, Lavretsky H, Kulkarni G, Jeste DV (2011). A tune in “a minor” can “b major”: a review of epidemiology, illness course, and public health implications of subthreshold depression in older adults. J Affect Disord.

[24] Bot M, Pouwer F, Ormel J, Slaets JPJ, de Jonge P (2010). Predictors of incident major depression in diabetic outpatients with subthreshold depression. Diabet Med.

[25] Hance M, Carney RM, Freedland KE, Skala J (1996). Depression in patients with coronary heart disease: a 12-month follow-up. Gen Hosp Psychiatry.

[26] Beck A, Crain AL, Solberg LI, Unutzer J, Glasgow RE, Maciosek MV, Whitebird R (2011). Severity of depression and magnitude of productivity loss. Ann Fam Med.

[27] Ruo B, Rumsfeld JS, Hlatky MA, Liu H, Browner WS, Whooley MA (2003). Depressive symptoms and health-related quality of life: the heart and soul study. Jama.

[28] Schram MT, Baan CA, Pouwer F (2009). Depression and quality of life in patients with diabetes: a systematic review from the European depression in diabetes (EDID) research consortium. Curr Diabetes Rev.

[29] van Zoonen K, Buntrock C, Ebert DD, Smit F, Reynolds CF, Beekman ATF, Cuijpers P (2014). Preventing the onset of major depressive disorder: a meta-analytic review of psychological interventions. Int J Epidemiol.

[30] Bower P, Gilbody S (2005). Stepped care in psychological therapies: access, effectiveness and efficiency: narrative literature review. Br J Psychiatry.

[31] van't Veer-Tazelaar PJ, van Marwijk HW, van Oppen P (2009). Stepped-care prevention of anxiety and depression in late life: a randomized controlled trial. Arch Gen Psychiatry.

[32] Dozeman E, van Marwijk HW, van Schaik DJ (2012). Contradictory effects for prevention of depression and anxiety in residents in homes for the elderly: a pragmatic randomized controlled trial. Int Psychogeriatr.

[33] van der Aa HP, van Rens GH, Comijs HC, Margrain TH, Gallindo-Garre F, Twisk JW, van Nispen RM. Stepped care for depression and anxiety in visually impaired older adults: multicentre randomised controlled trial. BMJ. 2015;351:h6127. https://doi.org/10.1136/bmj.h6127. 10.1136/bmj.h6127 PMC4655616 26597263

[34] Stoop C, Nefs G, Pommer A (2015). Effectiveness of a stepped care intervention for anxiety and depression in people with diabetes, asthma or COPD in primary care: a randomized controlled trial. J Affect Disord.

[35] van Beljouw IM, van Exel E, van de Ven PM (2015). Does an outreaching stepped care program reduce depressive symptoms in community-dwelling older adults? A randomized implementation trial. Am J Geriatr Psychiatry.

[36] van der Weele GM, de Waal MW, van den Hout WB (2012). Effects of a stepped-care intervention programme among older subjects who screened positive for depressive symptoms in general practice: the PROMODE randomised controlled trial. Age Ageing.

[37] Katon WJ, Lin EH, Von Korff M (2010). Collaborative care for patients with depression and chronic illnesses. N Engl J Med.

[38] Drummond MF, Sculpher MJ, Claxton K, et al. Methods for the economic evaluation of health care programmes. Oxford: Oxford university press; 2015.

[39] Van Dijk SE, Pols AD, Adriaanse MC, et al. Cost-effectiveness of a stepped-care intervention to prevent major depression in patients with type 2 diabetes mellitus and/or coronary heart disease and subthreshold depression: design of a cluster-randomized controlled trial. BMC Psychiatry. 2013;13(1):1–9. 10.1186/1471-244X-13-128 PMC3654943 23651614

[40] Kroenke K, Spitzer RL, Williams JB (2001). The PHQ-9: validity of a brief depression severity measure. J Gen Intern Med.

[41] Kroenke K, Spitzer RL (2002). The PHQ-9: a new depression diagnostic and severity measure. Psychiatr Ann.

[42] Lamers F, Jonkers CC, Bosma H (2008). Summed score of the patient health Questionnaire-9 was a reliable and valid method for depression screening in chronically ill elderly patients. J Clin Epidemiol.

[43] Sheehan DV, Lecrubier Y, Sheehan KH, Amorim P, Janavs J, Weiller E, Hergueta T, Baker R, Dunbar GC. The Mini-International Neuropsychiatric Interview (M.I.N.I.): the development and validation of a structured diagnostic psychiatric interview for DSM-IV and ICD-10. J Clin Psychiatry. 1998;59 (Suppl 20):22–33. 9881538

[44] Voordouw I, van Osch B, Terweij M. De cursus leven met een chronische ziekte. Handreiking voor coördinatoren en begeleiders. Utrecht: Trimbos-instituut; 2005.

[45] Schreuders B, van Marwijk H, Smit J, Rijmen F, Stalman W, van Oppen P (2007). Primary care patients with mental health problems: outcome of a randomised clinical trial. Br J Gen Pract.

[46] Mynors-Wallis L. Problem-solving treatment for anxiety and depression: a practical guide. Oxford: OUP Oxford; 2005.

[47] NHG-werkgroep Depressie. NHG-Standaard Depressie (M44). Utrecht: NHG; 2019.

[48] van Vliet IM, de Beurs E. Het Mini Internationaal Neuropsychiatrisch Interview (MINI). Een kort gestructureerd diagnostisch psychiatrisch interview voor DSM-IV- en ICD-10-stoornissen [The MINI-International Neuropsychiatric Interview. A brief structured diagnostic psychiatric interview for DSM-IV en ICD-10 psychiatric disorders]. Tijdschr Psychiatr. 2007;49(6):393-7. Dutch. 17614093

[49] Brooks R, Group E. EuroQol: the current state of play. Health Policy. 1996;37(1):53–72. 10.1016/0168-8510(96)00822-6. 10.1016/0168-8510(96)00822-6 10158943

[50] Lamers L, Stalmeier P, McDonnell J, Krabbe PF, van Busschbach J (2005). Measuring the quality of life in economic evaluations: the Dutch EQ-5D tariff. Ned Tijdschr Geneeskd.

[51] Zuithoff NP, Vergouwe Y, King M (2010). The patient health Questionnaire-9 for detection of major depressive disorder in primary care: consequences of current thresholds in a crosssectional study. BMC Fam Pract.

[52] Meader N, Mitchell AJ, Chew-Graham C, Goldberg D, Rizzo M, Bird V, Kessler D, Packham J, Haddad M, Pilling S (2011). Case identification of depression in patients with chronic physical health problems: a diagnostic accuracy meta-analysis of 113 studies. Br J Gen Pract.

[53] Statistics Netherlands, https://www.cbs.nl

[54] van Dongen JM, van Wier MF, Tompa E, Bongers PM, van der Beek AJ, van Tulder MW, Bosmans JE (2014). Trial-based economic evaluations in occupational health: principles, methods, and recommendations. J Occup Environ Med.

[55] Hakkaart-van Roijen L, Van Straten A, Donker M, et al. Manual Trimbos/iMTA questionnaire for costs associated with psychiatric illness (TiC-P). Rotterdam: iMTA, 2002.

[56] Hakkaart-van Roijen L, Tan S, Bouwmans C. Handleiding voor kostenonderzoek, methoden en standaard kostprijzen voor economische evaluaties in de gezondheidszorg. Geactualiseerde versie. Diemen: College voor Zorgverzekeringen; 2010.

[57] Dutch drug database G-standaard. The Hague: Stichting Z-index; 2009.

[58] Van Buuren S, Groothuis-Oudshoorn K (2011). Multivariate imputation by chained equations. J Stat Softw.

[59] White IR, Royston P, Wood AM (2011). Multiple imputation using chained equations: issues and guidance for practice. Stat Med.

[60] Rubin DB. Multiple imputation for nonresponse in surveys. Oxford: Wiley; 2004.

[61] Thompson SG, Barber JA (2000). How should cost data in pragmatic randomised trials be analysed?. Bmj.

[62] Fenwick E, O'Brien BJ, Briggs A (2004). Cost-effectiveness acceptability curves–facts, fallacies and frequently asked questions. Health Econ.

[63] Pols AD, Van Dijk SE, Bosmans JE (2017). Effectiveness of a stepped-care intervention to prevent major depression in patients with type 2 diabetes mellitus and/or coronary heart disease and subthreshold depression: a pragmatic cluster randomized controlled trial. PLoS One.

[64] Birch S, Gafni A (2002). On being NICE in the UK: guidelines for technology appraisal for the NHS in England and Wales. Health Econ.

[65] van't Veer-Tazelaar P, Smit F, van Hout H, van Oppen P, van der Horst H, Beekman A, van Marwijk H (2010). Cost-effectiveness of a stepped care intervention to prevent depression and anxiety in late life: randomised trial. Br J Psychiatry.

[66] Jonkers CC, Lamers F, Evers SM (2009). Economic evaluation of a minimal psychological intervention in chronically ill elderly patients with minor or mild to moderate depression: a randomized trial (the DELTA-study). Int J Technol Assess Health Care.

[67] American Diabetes Association. Standards of medical care in diabetes--2013.Diabetes Care. 2013;36(Suppl 1):S11–66. 10.2337/dc13-S011. 10.2337/dc13-S011 PMC3537269 23264422

[68] van Straten A, Hill J, Richards DA, Cuijpers P (2015). Stepped care treatment delivery for depression: a systematic review and meta-analysis. Psychol Med.

[69] Grochtdreis T, Brettschneider C, Wegener A, Watzke B, Riedel-Heller S, Härter M, König HH (2015). Cost-effectiveness of collaborative care for the treatment of depressive disorders in primary care: a systematic review. PLoS One.

[70] Lewis G, Araya R, Tang WK (2014). Prevention of anxiety and depression in Chinese: a randomized clinical trial testing the effectiveness of a stepped care program in primary care. J Affect Disord.

[71] van der Aa HP, van Rens GH, Bosmans JE (2017). Economic evaluation of stepped-care versus usual care for depression and anxiety in older adults with vision impairment: randomized controlled trial. BMC Psychiatry.

[72] Bosmans J, Dozeman E, van Marwijk HW (2014). Cost-effectiveness of a stepped care programme to prevent depression and anxiety in residents in homes for the older people: a randomised controlled trial. Int J Geriatr Psychiatry.

[73] Pols AD, Schipper K, Overkamp D (2017). Process evaluation of a stepped-care program to prevent depression in primary care: patients’ and practice nurses’ experiences. BMC Fam Pract.

[74] Briggs A. Economic evaluation and clinical trials: size matters. BMJ. 2000;321(7273):1362–3. 10.1136/bmj.321.7273.1362. 10.1136/bmj.321.7273.1362 PMC1119102 11099268

