# Supplementary material for: Cost-effectiveness of a stepped care program to prevent depression among primary care patients with diabetes mellitus type 2 and/or coronary heart disease and subthreshold depression in comparison with usual care
Source: BMC Psychiatry. 2021 Aug 13;21:402. doi: 10.1186/s12888-021-03367-z (PMC8361858; doi:10.1186/s12888-021-03367-z)
Supplement: Supplementary file 1 — Additional file 1. [file 12888_2021_3367_MOESM1_ESM.docx]

**Appendix A** International Classifications of Primary Care codes

| **ICPC code** | **Description** |
| --- | --- |
| T90 | Diabetes |
| T90.02 | Diabetes Mellitus type 2 |
| K74 | Angina pectoris |
| K74.01 | Instable angina pectoris |
| K74.02 | Stable angina pectoris |
| K75 | Acute myocardial infarction |
| K76 | Other/chronic ischemic disease |
| K76.01 | Coronary sclerosis |
| K76.02 | Previously experienced myocardial infarction (> 4 weeks ago) |

Appendix to:

Cost-effectiveness of a stepped care program to prevent depression among primary care patients with diabetes mellitus type 2 and/or coronary heart disease and subthreshold depression in comparison with usual care

S.E.M. van Dijk, MSc1, A.D. Pols, MD1,2, M.C. Adriaanse, PhD1, H.W.J. van Marwijk, PhD2,3,4, M.W. van Tulder, PhD1, J.E. Bosmans, PhD1*
